# Limitations of life-sustaining treatments in intensive care units in Croatia: a multicenter retrospective study

**DOI:** 10.3325/cmj.2024.65.373

**Published:** 2024-08

**Authors:** Diana Špoljar, Radovan Radonić, Zdravka Poljaković, Višnja Nesek, Marinko Vučić, Jasminka Peršec, Tatjana Kereš, Nenad Karanović, Krešimir Čaljkušić, Željko Župan, Igor Grubješić, Mia Golubić, Ana Jozepović, Bojana Nevajdić, Ana Borovečki, Dinko Tonković

**Affiliations:** 1 Community Health Center Zagreb – Center, Zagreb, Croatia; 2 Division of Intensive Care Medicine, Department of Internal Medicine, University Hospital Centre Zagreb, Zagreb, Croatia; 3 Department of Neurology, University Hospital Center Zagreb, Zagreb, Croatia; 4 Sveti Duh University Hospital Center, Zagreb, Croatia; 5 Sestre Milosrdnice University Hospital Center, Zagreb, Croatia; 6 Department of Anesthesiology, Reanimatology and Intensive Care Medicine, Dubrava University Hospital, Zagreb, Croatia; 7 Intensive Care Unit, Dubrava University Hospital, Zagreb, Croatia; 8 University Hospital Center Split, Split, Croatia; 9 Department of Anesthesiology, Reanimatology and Intensive Care Medicine, Faculty of Medicine, University of Rijeka, Rijeka, Croatia; 10 Zabok General Hospital, Zabok, Croatia; 11 School of Medicine, University of Zagreb, Zagreb, Croatia; 12 Marien Hospital Dusseldorf, Dusseldorf, Germany; 13 Andrija Štampar School of Public Health, School of Medicine, University of Zagreb, Zagreb, Croatia; 14 University Hospital Centre Zagreb, Zagreb, Croatia

## Abstract

**Aim:**

In order to gain insight into the current prevailing practices regarding the limitation of life-sustaining treatment in intensive care units (ICUs) in Croatia, we assessed the frequency of limitation and provision of certain treatment modalities, as well as the associated patient and ICU-related factors.

**Methods:**

A multicenter retrospective cross-sectional study was conducted in 17 ICUs in Croatia. We reviewed the medical records of patients deceased in 2017 and extracted data on demographic, clinical, and health care variables. A logistic regression analysis was conducted to determine the associations between these variables and treatment modalities.

**Results:**

The study enrolled 1095 patients (55% male; mean age 69.9 ± 13.7). Analgesia and sedation were discontinued before the patient’s death in 23% and 34% of the cases, respectively. Patients older than 71 years were less often mechanically ventilated (*P* < 0.001), and less frequently received inotropes and vasoactive therapy (*P* = 0.002) than younger patients. Patients hospitalized in the ICU for less than 7 days less frequently had discontinuation of mechanical ventilation and inotropes and vasoactive therapy than patients hospitalized for 8 days and longer (*P* < 0.001). Logistic regression analysis showed that ICU type was a crucial determinant, with multidisciplinary and surgical ICUs being associated with higher odds of intubation, mechanical ventilation, vasoactive and inotropic therapy, analgesia, and sedation.

**Conclusion:**

Older patients and those diagnosed with stroke and intracranial hemorrhage received fewer therapeutic modalities. All the observed treatment modalities were more frequently discontinued in patients who were hospitalized in the ICU for a prolonged time.

Limitation of life-sustaining treatments (LST) is a common aspect of work in intensive care units (ICUs). A part of the patients admitted to ICUs are considered palliative and end-of-life patients before admission. However, some of the patients become characterized as such during their stay in the ICU, as their health and medical conditions worsen. The decision to limit LST rests on the medical professionals’ assessment of the patient’s status and treatment futility. The frequency of decisions to limit LST in European countries has increased through the years (1,2). About 11% of all patients admitted to the ICU undergo some sort of limitation of LST (3). Withholding of LST is more common than withdrawing of LST, and treatment limitations are much more common in northern than southern Europe (3).

Patient characteristics and case mix vary in different types of ICUs. Surgical ICUs mainly admit younger patients requiring surgery, while medical and neurological ICUs more often admit patients burdened with chronic diseases and comorbidities. Older age and neurologic diseases, among other factors, have been associated with decisions to withdraw or withhold life support (4-6).

Croatia has not been included in international studies exploring issues regarding treatment of end-of-life patients in ICUs, nor were any such studies conducted on a national level. Recent research among ICU professionals in Croatia has shown that decisions to limit LST in end-of-life patients are not frequently made, even though most of the participants found that withholding and withdrawing of treatment was ethically acceptable (7).

The aim of this nationwide retrospective cross-sectional study was to assess the provision and limitation of certain treatment modalities in order to gain insight into the current practices regarding limitation of life-sustaining treatment in different types of ICUs in Croatia.

## Patients and methods

This cross-sectional study was conducted in 17 ICUs in 6 university hospital centers (UHC) of a tertiary level in Croatia from January to September 2019 (Zagreb UHC, Dubrava UHC, Sestre Milosrdnice UHC, Sveti Duh UHC, Rijeka UHC, Split UHC). The study was approved by the ethics committees of Zagreb University School of Medicine and of all the institutions involved in the research. Given the retrospective nature of the study and the absence of intervention, no prior informed consent was considered necessary.

### Patients

Four researchers reviewed the electronic database or paper records of each ICU and extracted the files of patients deceased in 2017. The list of deceased patients was provided by the ICU directors. A code was assigned to each patient to protect their anonymity.

Data were obtained on demographic variables, the main diagnosis at the time of death, comorbidities and pre-existing medical illnesses, length of hospital stay, length of stay in the ICU, provision and limitation of LST. Age was categorized into three groups: ≤45, 46-70, and ≥71 years. Length of hospitalization and ICU stay were categorized into groups: ≤7 days, 8-14 days, 15-29 days, and ≥30 days. It was possible to note multiple main and comorbid diagnoses at the time of death. The reasons for the limitation of therapeutic modalities were also noted if documented in the patient’s records.

The primary outcomes were the provision and discontinuation of the following treatment modalities: cardiopulmonary resuscitation and defibrillation (CPR), intubation, mechanical ventilation, inotropes and vasoactive therapy, antimicrobial therapy, analgesia, and sedation. The exposures included demographic data (age, sex), clinical variables (main diagnosis, comorbidities), and health care variables (length of hospitalization, length of stay in the ICU, type of ICU). Age, sex, length of hospitalization, length of stay in the ICU, and type of ICU were considered as predictors. Potential confounders included the severity of illness and comorbid conditions. The data collection methods were consistent across all participating ICUs, ensuring comparability. Information was extracted and recorded by trained researchers using a standardized form to minimize variability.

Efforts to address potential sources of bias included using a standardized data collection form to ensure uniformity, categorizing continuous variables (eg, age, length of stay) to facilitate comparison, employing logistic regression models to adjust for confounding variables such as age, sex, and ICU type, and ensuring complete data collection with no missing data reported.

### Statistical analysis

Data were summarized using descriptive statistics. The normality of the data distribution was tested with a Shapiro-Wilk test. Categorical variables are presented as absolute frequencies and percentages, and continuous variables as means and standard deviations. There were no missing data. Differences between the groups were evaluated with a χ^2^ test with Benjamini and Hochberg's false discovery rate *P* value correction method for multiple comparisons. *P* values lower than 0.05 were considered statistically significant. All tests were double-sided.

A logistic regression analysis was conducted to determine the associations between independent variables and treatment modalities. Independent variables were sex, age, length of hospitalization, length of ICU stay, and type of ICU. The reference category for ICU type was the medical ICU. Dependent variables included the provision of CPR, intubation, mechanical ventilation, vasoactive and inotropic therapy, antimicrobial therapy, analgesia, and sedation. Each logistic regression model included a constant term and independent variables. The results are presented as unadjusted frequencies and adjusted odds ratios with 95% confidence intervals for key interventions. Statistical analysis was performed using custom scripts written in Python 3.8. and statsmodels library. Subgroup analyses were conducted to examine differences in treatment modalities based on patient demographics and ICU types. Sensitivity analyses revealed no significant issues with multicollinearity, thus validating the inclusion of all predictor variables in the logistic regression models.

## RESULTS

### Patient characteristics

The study enrolled 1095 patients (mean age 69.9 ± 13.7; 55% male). The mean length of hospital stay was 12.1 ± 15.9 days, and the mean length of ICU stay was 7.5 ± 9.4 days. The majority of patients (54.8%) were admitted to the ICU from the Emergency Department, and only 4.9% were organ donors.

Overall, 45.6% of patients died of sepsis or septic shock, and 60.1% had a respiratory disease comorbidity. The same proportion of patients were admitted to surgical and neurological ICUs (29.4%) (Table 1).

**Table 1:** Patients’ characteristics

Characteristic		N	%
Sex	male	602	55
	female	493	45
Age (years)	≤45	68	6.2
	46-70	434	39.6
	≥71	593	54.2
Admitted from	emergency department	600	54.8
	other ICU od HDU	92	8.4
	ward	349	31.9
	other institution	52	4.7
ICU type	surgical	322	29.4
	medical	306	27.9
	neurological	322	29.4
	multidisciplinary	145	13.2
Length of hospitalization (days)	≤7	599	54.7
	8-14	206	18.8
	15-29	178	16.3
	≥30	105	9.6
Length of stay in the ICU (days)	≤7	758	69.2
	8-14	192	17.5
	15-29	105	9.6
	≥30	38	3.5
Main diagnosis at the time of death	sepsis, septic shock	499	45.6
	stroke and intracranial hemorrhage	435	39.7
	coma	248	22.6
	multiple-organ failure	155	14.2
	malignancy	114	10.4
	brain edema	49	4.5
	hemorrhagic shock	41	3.7
	cardiogenic shock	32	2.9
	multiple-trauma	23	2.1
	shock (unspecified)	18	1.6
	hypovolemic shock	7	0.6
	neurogenic shock	1	0.1
Comorbidities	respiratory	658	60.1
	cardiovascular	582	53.2
	arterial hypertension	495	45.2
	renal	410	37.4
	acute abdomen	367	33.5
	oncologic disease	293	26.8
	diabetes mellitus	275	25.1
	neurological	207	18.9
	psychiatric	104	9.5
	attempted suicide	11	1.0
Organ donor	yes	54	4.9
	no	1041	95.1

*Abbreviation: ICU – intensive care unit, HDU – high-dependency unit.

### Treatment modalities

Each treatment modality was employed in more than 50% of patients, except CPR, which was employed in 32% of patients. Intubation, mechanical ventilation, inotropes and vasoactive therapy, and antimicrobial therapy were provided to more than 70% of patients.

All the observed treatment modalities were at some point discontinued and reinstated in a small percentage of patients. Analgesia and sedation were discontinued before the patient’s death in 23% and 34% of cases, respectively (Table 2).

**Table 2:** Applied, discontinued, and reinstated treatment modalities

	No. (%) of patients with treatment		
Treatment	applied	discontinued and reinstated	discontinued before death (not reinstated)
Intubation	844 (77.1)	70 (6.4)	42 (3.9)
Mechanical ventilation	830 (75.8)	94 (8.6)	68 (6.2)
Inotropes and vasoactive therapy	814 (74.3)	123 (11.2)	131 (12.0)
Antimicrobial therapy	923 (84.3)	32 (2.9)	54 (4.9)
Analgesia	702 (64.1)	116 (10.6)	258 (23.7)
Sedation	593 (54.2)	128 (11.7)	372 (34.1)

The reasons for discontinuation of treatments were not noted, except in two cases where documents stated that a group of physicians had agreed on the futility of further treatment.

### Differences in the provision of treatment modalities

The analysis included age, sex, length of stay in the ICU, type of ICU, and 5 most frequent main diagnoses – sepsis and septic shock, stroke and intracranial hemorrhage, coma, multiple-organ failure (MOF), and malignancy.

Patients older than 71 years were less often intubated and mechanically ventilated (*P* < 0.001) and less frequently received inotropes and vasoactive therapy (*P* = 0.002), analgesia (*P* = 0.01), and sedation (*P* < 0.001) than younger patients.

Patients hospitalized in the ICU for fewer than 7 days more frequently received CPR measures than those hospitalized for 8-30 days (*P* = 0.043). They were less frequently intubated and mechanically ventilated (*P* = 0.031), and less frequently received antimicrobial therapy (*P* = 0.006), analgesia (*P* < 0.001), and sedation (*P* < 0.001) than patients hospitalized for 8 or more days. They also less frequently received inotropes and vasoactive therapy than patients who spent between 15 and 29 days in the ICU (*P* < 0.001).

Compared with female patients, male patients were more often intubated and mechanically ventilated (*P* < 0.001), more frequently received inotropes and vasoactive therapy (*P* = 0.002), antimicrobial therapy (*P* = 0.044), analgesia (*P* = 0.006), and sedation (*P* < 0.001).

Patients hospitalized in medical ICUs more frequently received CPR measures than those hospitalized in other types of ICUs (*P* = 0.015). Those hospitalized in surgical ICUs were more often intubated and mechanically ventilated, received inotropes and vasoactive therapy, analgesia, and sedation than patients in neurological and multidisciplinary ICUs (*P* < 0.001 for all). Patients in neurological ICUs less frequently received antimicrobial therapy than patients hospitalized in other types of ICUs (*P* < 0.001).

Patients diagnosed with sepsis and MOF received more CPR measures (*P* = 0.04), more often were intubated and mechanically ventilated (*P* = 0.27), received inotropes and vasoactive therapies (*P* < 0.001), and received antimicrobial therapies (*P* < 0.001) than patients with stroke and intracranial hemorrhage or malignant diseases (Table 3).

**Table 3:** Differences in the provision of treatment modalities and discontinuation before patient’s death

		Total N of patients in the group	CPR measures	Intubation		Mechanical ventilation		Inotropes and vasoactive therapy		Antimicrobial therapy		Analgesia		Sedation	
			received n (%)	received n (%)	discont-inued before death n (%)	received n (%)	discont-inued before death n (%)	received n (%)	discont-inued before death n (%)	received n (%)	discont-inued before death n (%)	received n (%)	discont-inued before death n (%)	received n (%)	Discont-inued before death N (%)
Age (years)	≤45	68	23 (33.8)	67 (98.5)	2 (3.0)	68 (100)	2 (2.9)	59 (86.8)	11 (18.6)	62 (91.2)	5 (8.1)	51 (75.0)	19 (37.3)	54 (79.4)	32 (59.3)
	46-70	434	151 (34.8)	378 (87.1)	16 (4.2)	370 (85.3)	26 (7.0)	361 (83.2)	52 (14.4)	374 (86.2)	19 (5.1)	313 (72.1)	114 (36.4)	266 (61.3)	170 (63.9)
	≥71	593	177 (29.8)	399 (67.3)	24 (6.0)	392 (66.1)	40 (10.2)	394 (66.4)	68 (17.3)	487 (82.1)	30 (6.2)	338 (57.0)	125 (37.0)	273 (46.0)	170 (62.3)
Sex	male	602	193 (32.1)	489 (81.2)	16 (3.3)	483 (80.2)	29 (6.0)	470 (78.1)	74 (15.7)	520 (86.4)	32 (6.2)	408 (67.8)	154 (37.7)	369 (61.3)	227 (61.5)
	female	493	158 (32.0)	355 (72.0)	26 (7.3)	347 (70.4)	39 (11.2)	344 (69.8)	57 (16.6)	403 (81.7)	22 (5.5)	294 (59.6)	104 (35.4)	224 (45.4	145 (64.7)
Length of stay in the ICU (days)	≤7	758	269 (35.5)	554 (73.1)	11 (2.0)	542 (71.5)	16 (3.0)	539 (71.1)	39 (7.2)	597 (78.8)	17 (2.8)	412 (54.4)	119 (28.9)	329 (43.4)	161 (48.9)
	8-14	192	48 (25.0)	158 (82.3)	17 (10.8)	156 (81.2)	24 (15.4)	148 (77.1)	41 (27.7)	185 (96.4)	12 (6.5)	162 (84.4)	76 (46.9)	137 (71.4)	108 (78.8)
	15-29	105	24 (22.9)	96 (91.4)	11 (11.5)	95 (90.5)	20 (21.1)	93 (88.6)	38 (40.9)	101 (96.2)	16 (15.8)	91 (86.7)	45 (49.5)	91 (86.7)	73 (80.2)
	≥30	38	9 (23.7)	35 (92.1)	3 (8.6)	36 (94.7)	7 (19.4)	32 (84.2)	12 (37.5)	38 (100.0)	9 (23.7)	36 (94.7)	18 (50.0)	35 (92.1)	29 (82.9)
ICU type	surgical	322	75 (23.3)	313 (97.2)	13 (4.2)	312 (96.9)	23 (7.4)	309 (96.0)	44 (14.2)	292 (90.7)	22 (7.5)	277 (86.0)	81 (29.2)	239 (71.4)	151 (65.7)
	medical	306	154 (50.3)	223 (72.9)	17 (7.6)	216 (70.6)	21 (9.7)	235 (76.8)	38 (16.2)	273 (89.2)	15 (5.5)	129 (42.2)	53 (41.1)	129 (42.2)	73 (56.6)
	neurological	322	68 (21.2)	168 (52.2)	11 (6.5)	162 (50.3)	15 (9.3)	136 (42.4)	32 (23.5)	221 (68.6)	15 (6.8)	175 (54.3)	77 (44.0)	122 (37.9)	81 (66.4)
	multi-disciplinary	145	54 (37.2)	140 (96.6)	1 (0.7)	140 (96.6)	9 (6.4)	134 (92.4)	17 (12.7)	137 (94.5)	2 (1.5)	121 (83.4)	47 (38.8)	112 (77.2)	67 (59.8)
Main diagno-sis	sepsis	499	188 (37.7)	428 (85.8)	23 (5.4)	424 (85.0)	37 (8.7)	449 (90.0)	62 (13.8)	488 (97.8)	20 (4.1)	362 (72.5)	112 (30.9)	307 (61.5)	183 (59.6)
	stroke and intracranial hemorrhage	435	91 (20.9)	287 (66.0)	13 (4.5)	279 (64.1)	21 (7.5)	249 (57.2)	54 (21.7)	321 (73.8)	22 (6.9)	252 (57.9)	128 (50.8)	210 (48.3)	154 (73.3)
	coma	248	65 (26.2)	198 (79.8)	10 (5.1)	193 (77.8)	12 (6.2)	171 (69.0)	39 (22.8)	191 (77.0)	14 (7.3)	146 (58.9)	79 (54.1)	124 (50.0)	99 (79.8)
	multiple-organ failure	155	61 (39.4)	136 (87.7)	3 (2.2)	134 (86.5)	10 (7.5)	147 (94.8)	19 (12.9)	152 (98.0)	5 (3.3)	113 (72.9)	30 (26.5)	85 (54.8)	45 (52.9)
	malignancy	114	29 (25.4)	77 (67.5)	3 (3.9)	76 (66.7)	7 (9.2)	73 (64.0)	12 (16.4)	95 (83.3)	11(11.6)	84 (73.7)	19 (22.6)	61 (53.5)	35 (57.4)

*Abbreviations: ICU – intensive care unit; CPR – cardiopulmonary resuscitation.

### Differences in discontinuation of treatment modalities before the patient’s death

Since the reasons for discontinuation of treatments were not noted, we could not evaluate the differences in the limitation of LST. However, we evaluated the differences between groups in treatment modalities that were discontinued but not reinstated before the patient’s death.

The longer the hospitalization in the ICU, the more frequent the discontinuation of all treatment modalities. Patients hospitalized in the ICU for less than 7 days less frequently had discontinuation of intubation than patients hospitalized for 8-29 days (*P* < 0.001), and less frequently had discontinuation of mechanical ventilation and of inotropes and vasoactive therapy than those hospitalized for 8 days or longer (*P* < 0.001 for all groups). Patients hospitalized for less than 7 days less frequently had discontinuation of antimicrobial treatment than those hospitalized 8-14 days (*P* = 0.047) and 15 or more days (*P* < 0.001). Patients hospitalized in the ICU for less than 7 days less frequently had discontinuation of analgesia than patients hospitalized for 8-14 (*P* < 0.001), 15-29 (*P* = 0.001), and more than 30 days (*P* = 0.029) and less frequently had discontinuation of sedation than patients hospitalized for 8-29 (*P* < 0.001) and more than 30 days (*P* = 0.001).

Discontinuation of intubation was more frequent in medical ICUs than in multidisciplinary ICUs (*P* = 0.041), while discontinuation of analgesia was more frequent in neurological than in surgical ICUs (*P* = 0.011).

Analgesia was more frequently discontinued in patients diagnosed with stroke and intracranial hemorrhage and with coma than in patients diagnosed with sepsis, MOF, and malignancy (*P* < 0.001 for all). Sedation was also more frequently discontinued in patients with stroke and intracranial hemorrhage than in patients with sepsis (*P* = 0.004), MOF (*P* = 0.004), and malignancy (*P* = 0.043).

### Logistic regression analysis

Logistic regression analysis showed that the most prominent independent variables were length of ICU stay, type of ICU, and age.

Each additional day in the ICU was associated with a 3.7% decrease in the odds of CPR provision (*P* = 0.002), 5.4% increase in the odds of provision of intubation (*P* = 0.009), 6.3% increase in the odds of provision of mechanical ventilation (*P* = 0.003), 3.9% increase in the odds of provision of vasoactive and inotropic therapy (*P* = 0.007), 26.0% in the odds of provision of antimicrobial therapy (*P* < 0.001), 18.2% in the odds of provision of analgesia (*P* < 0.001), and 14.4% in the odds of provision of sedation (*P* < 0.001).

In comparison with patients in medical ICUs, patients in multidisciplinary ICUs had 41.5% lower odds of provision of CPR (*P* = 0.011); however, they were 10.78 times more likely to be intubated (*P* < 0.001), 12.01 times more likely to be mechanically ventilated (*P* < 0.001), 4.05 times more likely to receive vasoactive and inotropic therapies (*P* < 0.001), 8.67 more likely to receive analgesia (*P* = 0.003), and 5.40 times more likely to receive sedation (*P* < 0.001).

Patients in neurological ICUs had 72.8% lower odds of undergoing CPR (*P* < 0.001). They were 72.8% less likely to be intubated (*P* < 0.001), 59.0% less likely to be mechanically ventilated (*P* < 0.001), 78.2% less likely to receive vasoactive and inotropic therapies (*P* < 0.001), 78.2% less likely to receive antimicrobial therapy (*P* < 0.001), and had 1.71 times higher odds of receiving analgesia (*P* = 0.003). Patients in surgical ICUs had 8.93 times higher odds of receiving analgesia (*P* < 0.001) and 3.31 times higher odds of receiving sedation (*P* < 0.001) than patients in medical ICUs.

Older patients had slightly lower odds of undergoing CPR (*P* = 0.050). Each additional year of age decreased the odds of provision of intubation by 6.6% (*P* < 0.001), mechanical ventilation by 6.6% (*P* < 0.001), vasoactive and inotropic therapies by 3.9% (*P* < 0.001), analgesia by 2.0% (*P* < 0.001), and sedation by 3.1%. More details on the results of logistic regression analysis are provided in Supplemental Tables 1-7(Supplementary Tables).

## DISCUSSION

This is the first retrospective research about LST limitation in Croatian ICUs. The study showed that a small percentage of patients had some treatment modalities discontinued. CPR measures were less employed than other treatment modalities, and older patients and those diagnosed with stroke and intracranial hemorrhage received less therapeutic modalities. Patients treated in surgical ICUs had higher odds of receiving intubation, mechanical ventilation, vasoactive and inotropic therapy, analgesia, and sedation than patients in neurological ICUs. All the observed treatment modalities were more frequently discontinued in patients who were hospitalized in the ICU for a prolonged time.

This research showed that some treatment modalities were discontinued before the patient's death; however, no written explanation for these decisions was found. Medical records of only 2 patients contained a document stating that a group of physicians had agreed on the futility of further treatment. Since the reasons behind the other treatment discontinuations are not known, we can only assume that certain discontinuations of treatment modalities were in fact acts of LST limitation in palliative patients.

Similarly to our results, a cross-sectional study in Croatian ICUs by Špoljar et al found that decisions to limit LST were conveyed among the members of the medical team in verbal, rather than written, form (7). Evidently, written instructions or other notes pertaining to end-of-life issues are still not a standard part of medical records in Croatia.

The Croatian Ministry of Health, in its guidelines on improving quality of palliative care in ICUs, recommended the development of a standardized hospital form for documenting all decisions related to application or limitation of LST. The Ministry also recommended that such decisions should be documented with a time stamp and physician’s signature in patients’ medical records (8).

Considering that the Croatian law does not allow limitation of LST in end-of-life patients, and that physicians can be criminally persecuted if they do limit it, it is not surprising that decisions to limit LST are not noted in medical records (9-11). In other studies as well, the level of documenting decisions on LST limitation was often low, fragmentary, or ambiguous (6,12,13). Additionally, end-of-life legislation was associated with limitation decisions, which suggests that legal recognition of end-of-life issues may amplify appropriate end-of-life decisions (3,14). This indicates that perhaps it is time for Croatian legislation to be revised.

In this study, CPR measures were less employed than other treatment modalities. Previously, it was reported that 88% of ICU physicians in Croatia found do-not-attempt CPR decisions ethically acceptable (7). In a prospective study among patients who died in the ICU or were discharged in terminal condition, about half of the limitations were attributed to do-not-resuscitate orders (15). Cook et al found an increase in do-not-resuscitate orders over the course of the patient’s stay in the ICU (16), and our research showed that each additional day in the ICU was associated with a 3.7% decrease in the odds of CPR being performed. Both studies indicate that a prolonged ICU stay is linked to fewer provisions of CPR measures.

This study showed consistently lower odds of receiving intubation, mechanical ventilation, and sedation in older patients. Previously, older age was associated with decisions to withdraw or withhold life support in trauma and non-trauma patients (5,17-21). Guidet et al found that LST in intensive care patients aged ≥80 years was less frequently limited in eastern and southern than in northern European countries (22). LST was also more frequently limited in countries with high GDP and less frequently in religious countries (22). Older patients, compared with younger ones, have reduced functional reserves, more comorbidities, and greater use of chronic medications. Advanced age can be a significant independent risk factor for mortality, especially in ICU patients older than 75 (23).

Most in-hospital deaths of patients with neurological conditions and diseases result from an LST limitation (4,24). Acute neurologic disease was connected to an increase in LST limitations (3,6). Our research showed that patients with stroke and intracranial hemorrhage less frequently received CPR, were less frequently intubated and mechanically ventilated, and less frequently received inotropes and vasoactive and antimicrobial therapies than patients with sepsis and MOF. Moreover, in these patients analgesia and sedation were more frequently discontinued.

In a previous study, mechanical ventilation was less frequently limited among surgery than neurology/neurosurgery patients (25). In our study, patients hospitalized in surgical ICUs were more often intubated and mechanically ventilated, received more inotropes and vasoactive therapy, and more analgesia and sedation than patients hospitalized in neurological and multidisciplinary ICUs. The results from a cross-sectional study conducted in Croatian ICUs (7) point in the same direction, showing that medical professionals working in neurological ICUs were more prone to limiting mechanical ventilation and hydration, and to removing the endotracheal tube. These results are not surprising considering the difference in patient population and case mix in various types of ICUs. Patients admitted to medical and neurological ICUs often have chronic conditions and multiple comorbidities, while surgical ICUs mainly admit younger patients requiring surgery.

A study conducted in eight Greek multidisciplinary ICUs also showed that patients who received full support were more likely to have surgical rather than medical conditions, and that patients admitted with a neurologic diagnosis were more likely to undergo limitation of treatment (12). In a study conducted in medical-surgical ICUs in Spain, a decision to withhold or withdraw LST was made in 65% of patients dying of non-traumatic coma and 36% of patients dying of sepsis and multiple organ dysfunction syndrome (26). A systematic review of critically ill patients in all types of ICUs in the United States also found that surgical patients were more likely to die with full interventions, and that LST limitation preceding death was more likely in medical patients (21).

According to a cross-sectional survey on a nationally representative Croatian sample, the most important characteristic of a “good death” was the absence of pain (27). Nonetheless, in this study, analgesia and sedation were discontinued before death in 23% and 34% of patients, respectively. Discontinuation of analgesia was more frequent in neurological than in surgical ICUs, and both analgesia and sedation were more frequently discontinued in patients diagnosed with stroke, intracranial hemorrhage, or coma than in patients with other diagnoses. Care for end-of-life patients encompasses the alleviation of pain and suffering. Medical, surgical, and trauma ICU patients routinely experience pain, both at rest and with routine ICU care (28,29). Many guidelines highlight the importance of alleviating pain, anxiety, and other uncomfortable symptoms even when such treatment may hasten patients’ death (8,30). Special consideration should be paid to patients who have no prospect of cure, like burn victims, and who should experience more comfortable and peaceful end-of-life care. Recognizing the inevitability of death opens a way to a more humane comfort care for such patients (31). Some neurological conditions affecting the central nervous system may exclude the need for sedation. However, this research showed that a relatively high percentage of patients diagnosed with other conditions died without receiving analgesia and sedation, which raises the question of adequacy of comfort care. For patients who cannot self-report their levels of pain, pain can be assessed with standardized pain observation tools or with the help of family members (8,32). Clearly defined protocols and guidelines provide the medical team with support in all aspects of treatment and can be associated with significant cost savings (31,33). Further research with a specific focus on comfort care should be conducted to clarify the results of this research.

This research has several limitations. All included ICUs were in tertiary-level university hospitals, so the results may not be representative of ICUs in general hospitals in Croatia. The researchers extracting the data from the medical records were not critical care specialists. Furthermore, most of the medical records were in paper form, some were disorganized, and missing certain documents. The handwriting on the treatment lists was not always easily legible, which might have led to misinterpretation. As previously mentioned, there were no written explanations for discontinuation of treatment modalities, therefore the differences in LST limitation could not be assessed.

Further research is needed to explore all aspects of the treatment of end-of-life patients, but especially to elucidate the reasons behind the discontinuation of treatment modalities and to evaluate the genuine frequency of LST limitation.
