# Supplementary material for: Limitations of life-sustaining treatments in intensive care units in Croatia: a multicenter retrospective study
Source: Croat Med J. 2024 Aug;65(4):373–82. doi: 10.3325/cmj.2024.65.373 (PMC11399720; doi:10.3325/cmj.2024.65.373)
Supplement: Supplementary Tables [file CroatMedJ_65_s003.pdf]

Supplemental Table 1. Logistic regression analysis results for provision of CPR
Coefficients

					Wald Test			95% Confidence interval (odds ratio scale)	
	Estimate	Standard Error	Odds Ratio	z	Wald Statistic	df	p	Lower bound	Upper bound
(Intercept)	0.889	0.403	2.434	2.209	4.880	1	0.027	1.106	5.357
Sex M	0.045	0.141	1.046	0.322	0.104	1	0.747	0.794	1.379
Age	-0.010	0.005	0.990	-1.961	3.846	1	0.050	0.980	1.000
Hospitalization length	0.004	0.006	1.004	0.582	0.338	1	0.561	0.992	1.015
ICU stay length	-0.037	0.012	0.963	-3.155	9.956	1	0.002	0.941	0.986
Multidisciplinary ICU	-0.536	0.211	0.585	-2.543	6.469	1	0.011	0.387	0.884
Neurological ICU	-1.301	0.181	0.272	-7.200	51.842	1	< .001	0.191	0.388
Surgical ICU	-1.199	0.180	0.302	-6.660	44.351	1	< .001	0.212	0.429

Supplemental Table 2. Logistic regression analysis results for provision of intubation

Coefficients

					Wald Test			95% Confidence interval (odds ratio scale)	
	Estimate	Standard Error	Odds Ratio	z	Wald Statistic	df	p	Lower bound	Upper bound
(Intercept)	5.632	0.703	279.312	8.018	64.281	1	< .001	70.489	1106.774
Sex M	0.115	0.180	1.122	0.639	0.409	1	0.523	0.789	1.596
Age	-0.069	0.009	0.934	-7.776	60.460	1	< .001	0.918	0.950
Hospitalization length	0.000	0.012	1.000	0.012	1.549×10^{-4}	1	0.990	0.976	1.025
ICU stay length	0.053	0.020	1.054	2.604	6.782	1	0.009	1.013	1.097
Multidisciplinary ICU	2.378	0.483	10.782	4.921	24.221	1	< .001	4.183	27.796
Neurological ICU	-0.896	0.187	0.408	-4.781	22.853	1	< .001	0.283	0.589
Surgical ICU	2.524	0.373	12.481	6.775	45.901	1	< .001	6.013	25.905

Supplemental Table 3. Logistic regression analysis results for provision of mechanical ventilation

Coefficients

					Wald Test			95% Confidence interval (odds ratio scale)	
	Estimate	Standard Error	Odds Ratio	z	Wald Statistic	df	p	Lower bound	Upper bound
(Intercept)	5.425	0.688	227.071	7.885	62.173	1	< .001	58.951	874.642
Sex M	0.178	0.178	1.195	0.999	0.998	1	0.318	0.843	1.695
Age	-0.068	0.009	0.934	-7.840	61.464	1	< .001	0.918	0.950
Hospitalization length	-0.001	0.012	0.999	-0.076	0.006	1	0.939	0.976	1.023
ICU stay length	0.061	0.020	1.063	2.980	8.883	1	0.003	1.021	1.106
Multidisciplinary ICU	2.485	0.483	12.005	5.148	26.506	1	< .001	4.661	30.923
Neurological ICU	-0.892	0.187	0.410	-4.781	22.857	1	< .001	0.284	0.591
Surgical ICU	2.507	0.356	12.268	7.033	49.457	1	< .001	6.100	24.672

Supplemental Table 4. Logistic regression analysis results for provision of vasoactive and intropic therapy

Coefficients

					Wald Test			95% Confidence interval (odds ratio scale)	
	Estimate	Standard Error	Odds Ratio	z	Wald Statistic	df	p	Lower bound	Upper bound
(Intercept)	3.786	0.584	44.096	6.486	42.063	1	< .001	14.043	138.466
Sex M	0.262	0.171	1.300	1.530	2.342	1	0.126	0.929	1.818
Age	-0.039	0.007	0.961	-5.345	28.567	1	< .001	0.948	0.975
Hospitalization length	-0.014	0.009	0.986	-1.579	2.492	1	0.114	0.970	1.003
ICU stay length	0.039	0.014	1.039	2.690	7.236	1	0.007	1.011	1.069
Multidisciplinary ICU	1.398	0.361	4.047	3.868	14.963	1	< .001	1.993	8.218
Neurological ICU	-1.540	0.185	0.214	-8.308	69.025	1	< .001	0.149	0.308
Surgical ICU	1.951	0.322	7.035	6.051	36.610	1	< .001	3.739	13.234

Supplemental Table 5. Logistic regression analysis results for provision of antimicrobial therapy

Coefficients

					Wald Test			95% Confidence interval (odds ratio scale)	
	Estimate	Standard Error	Odds Ratio	z	Wald Statistic	df	p	Lower bound	Upper bound
(Intercept)	1.153	0.594	3.169	1.941	3.768	1	0.052	0.989	10.156
Sex M	0.111	0.192	1.118	0.581	0.338	1	0.561	0.768	1.627
Age	-0.004	0.007	0.996	-0.561	0.314	1	0.575	0.982	1.010
Hospitalization length	0.051	0.021	1.052	2.443	5.968	1	0.015	1.010	1.096
ICU stay length	0.231	0.045	1.260	5.147	26.492	1	< .001	1.154	1.376
Multidisciplinary ICU	0.621	0.419	1.861	1.483	2.199	1	0.138	0.819	4.229
Neurological ICU	-1.523	0.237	0.218	-6.428	41.321	1	< .001	0.137	0.347
Surgical ICU	-0.156	0.283	0.856	-0.550	0.302	1	0.582	0.491	1.491

Supplemental Table 6. Logistic regression analysis results for provision of analgesia

Coefficients

					Wald Test			95% Confidence interval (odds ratio scale)	
	Estimate	Standard Error	Odds Ratio	z	Wald Statistic	df	p	Lower bound	Upper bound
(Intercept)	0.204	0.460	1.226	0.444	0.197	1	0.657	0.498	3.020
Sex M	0.072	0.151	1.075	0.476	0.227	1	0.634	0.799	1.445
Age	-0.020	0.006	0.980	-3.358	11.279	1	< .001	0.969	0.992
Hospitalization length	-0.017	0.007	0.983	-2.278	5.187	1	0.023	0.969	0.998
ICU stay length	0.167	0.020	1.182	8.359	69.880	1	< .001	1.136	1.229
Multidisciplinary ICU	2.160	0.272	8.673	7.938	63.013	1	< .001	5.088	14.785
Neurological ICU	0.537	0.177	1.710	3.023	9.139	1	0.003	1.208	2.422
Surgical ICU	2.190	0.215	8.932	10.184	103.711	1	< .001	5.861	13.614

Supplemental Table 7. Logistic regression analysis results for provision of sedation

Coefficients

					Wald Test			95% Confidence interval (odds ratio scale)	
	Estimate	Standard Error	Odds Ratio	z	Wald Statistic	df	P	Lower bound	Upper bound
(Intercept)	0.918	0.438	2.505	2.094	4.386	1	0.036	1.061	5.916
Sex M	0.404	0.145	1.498	2.793	7.801	1	0.005	1.128	1.988
Age	-0.032	0.006	0.969	-5.584	31.180	1	< .001	0.958	0.980
Hospitalization length	-0.007	0.007	0.993	-0.955	0.913	1	0.339	0.980	1.007
ICU stay length	0.135	0.017	1.144	8.055	64.888	1	< .001	1.107	1.182
Multidisciplinary ICU	1.686	0.248	5.397	6.788	46.082	1	< .001	3.317	8.782
Neurological ICU	-0.168	0.183	0.845	-0.922	0.849	1	0.357	0.591	1.209
Surgical ICU	1.198	0.189	3.313	6.348	40.299	1	< .001	2.289	4.796
